# Zinc release from thapsigargin/IP3-sensitive stores in cultured cortical neurons

**DOI:** 10.1186/1750-2187-5-5

**Published:** 2010-05-26

**Authors:** Christian J Stork, Yang V Li

**Affiliations:** 1 Molecular and Cellular Biology Program, Ohio University, Athens, OH 45701, USA; 2 Department of Biomedical Science, Ohio University, Athens, OH 45701, USA; 3 Neuroscience Program, Ohio University, Athens, OH 45701, USA

## Abstract

**Background:**

Changes in ionic concentration have a fundamental effect on numerous physiological processes. For example, IP_3_-gated thapsigargin sensitive intracellular calcium (Ca^2+^) storage provides a source of the ion for many cellular signaling events. Less is known about the dynamics of other intracellular ions. The present study investigated the intracellular source of zinc (Zn^2+^) that has been reported to play a role in cell signaling.

**Results:**

In primary cultured cortical cells (neurons) labeled with intracellular fluorescent Zn^2+ ^indicators, we showed that intracellular regions of Zn^2+ ^staining co-localized with the endoplasmic reticulum (ER). The latter was identified with ER-tracker Red, a marker for ER. The colocalization was abolished upon exposure to the Zn^2+ ^chelator TPEN, indicating that the local Zn^2+ ^fluorescence represented free Zn^2+ ^localized to the ER in the basal condition. Blockade of the ER Ca^2+ ^pump by thapsigargin produced a steady increase of intracellular Zn^2+^. Furthermore, we determined that the thapsigargin-induced Zn^2+ ^increase was not dependent on extracellular Ca^2+ ^or extracellular Zn^2+^, suggesting that it was of intracellular origin. The applications of caged IP_3 _or IP_3_-3Kinase inhibitor (to increase available IP_3_) produced a significant increase in intracellular Zn^2+^.

**Conclusions:**

Taken together, these results suggest that Zn^2+ ^is sequestered into thapsigargin/IP_3_-sensitive stores and is released upon agonist stimulation.

## Background

Zn^2+ ^is an important structural and functional component in many cellular proteins and enzymes. As such, Zn^2+ ^levels are normally tightly regulated, limiting the extent of cytosolic labile (or free) Zn^2+ ^concentrations [1,2]. For example, levels of free Zn^2+ ^are several orders of magnitude less than that of Ca^2+ ^[3]. Zn^2+ ^may act as a cellular messenger in physiological and cytotoxic signaling, and the changes in Zn^2+ ^homeostasis have a fundamental effect in cell function [4,5]. Many studies have shown the accumulation of excessive Zn^2+ ^to precede cell death or neurodegeneration in response to cytotoxic stress [6,7]. To characterize Zn^2+^-mediated signaling pathways or Zn^2+^-induced cytotoxicity, it is important to determine the source(s) of intracellular free Zn^2+ ^in response to specific stimuli or injury.

The endoplasmic reticulum (ER) is an intracellular organelle that has been shown to sequester Ca^2+ ^from the cytosol by means of sarcoplasmic/endoplasmic reticulum Ca^2+^-ATPase (SERCA) or so-called endoplasmic Ca^2+ ^pump [8]. This sequestered Ca^2+ ^can be released into the cytosol upon a variety of stimuli including inositol 1,4,5-trisphosphate (IP_3_). It is IP_3 _that mobilized Ca^2+ ^from the ER Ca^2+ ^store following interaction with specific IP_3 _receptors (IP_3_R). A commonly used tool in studying Ca^2+ ^homeostasis is thapsigargin, a plant derived compound that specifically inhibits SERCA activity [9]. By blocking the ability of the cell to pump Ca^2+ ^into the ER, thapsigargin causes these stores to become depleted and thereby raise the cytosolic Ca^2+ ^concentration.

While the mechanisms responsible for regulating Zn^2+ ^homeostasis are not well established, available data support that, like Ca^2+^, intracellular Zn^2+ ^levels are determined by the interaction of membrane Zn^2+ ^transporters and cytoplasmic Zn^2+ ^buffers [4,10]. The present study investigates the intracellular source of free Zn^2+^, particularly, if thapsigargin can trigger the release of Zn^2+^. This possibility is supported by recent evidence that Zn^2+ ^can be released from intracellular sources upon stimulation [11-13]. Our results show that Zn^2+ ^is released from thapsigargin-sensitive and IP_3_R-mediated stores.

## Methods

### Primary Cell Culture

Pregnant Sprague-Dawley rats (E17-E18) were anaesthetized with CO_2 _and the fetuses were removed and placed in ice-cold Hank's Balanced Salt Solution without Ca^2+ ^or Mg^2+ ^(HBSS). The brains of fetuses were removed and placed into cold HBSS for further dissection. Using a dissecting microscope and blunt dissection, the meninges were gently separated away. The cerebral cortex was then removed and each cortical hemisphere was cut into four pieces and trypsinized in HBSS at 37°C. Following trypsinization, cells were separated by trituration through the opening of a fire polished Pasteur pipette. The suspensions were then passed through a 70 μm cell strainer. The dissociated cells were added to the bottom of 35mm glass-bottomed petri dishes previously coated with polyethyleneimine (50% solution, Sigma, St. Louis) diluted 1:1000 in borate buffer. The cortical neurons were then allowed to attach to the surface at 37°C, 5% CO_2 _in 2 ml of MEM solution (Gibco, BRL) supplemented with 10% (v/v) heat-inactivated fetal bovine serum. After 3-6 hrs, solutions were replaced with fresh supplemented MEM which was later replaced (24 hrs) with Neurobasal medium (Gibco, BRL) supplemented with 2% B-27.

### Fluorescence Microscopy

All imaging experiments were performed in HEPES medium containing the following (in mM): 130 NaCl, 5 KCl, 8 MgSO_4_, 1 Na_2_HPO_4_, 25 Glucose, 20 HEPES, 1 Na-Pyruvate; pH adjusted to 7.4. Cortical cells grown in glass-bottomed petri dishes were washed with fresh HEPES medium. The cells were then incubated at 37°C for 30 min with ER-Tracker Red (Molecular Probes, Carlsbad, CA) and Newport Green (Molecular Probes, Carlsbad, CA) or ZinPyr-1 (Neurobiotex, Galveston, TX). Cells were incubated with 10 μM of the specified fluorescent Zn^2+ ^indicator either alone or in conjunction with ER-Tracker red for 30 min then were washed 3x with fresh HEPES medium and placed into a custom 35mm stage adapter and continuously perfused with fresh HEPES medium on the stage of a Zeiss LSM 510 (confocal) microscope. Cultures were examined using a Plan-Neofluar 100x/1.3 NA oil immersion objective. For ER-Tracker Red excitation was done with a HeNe Laser line of 543nm and an LP560nm emission filter. Newport Green and ZinPyr-1 were imaged using an Argon Laser line of 488nm for excitation and a BP505-550nm emission filter. Separate fluorescent channels were employed for each indicator, and channels were scanned sequentially to minimize crosstalk. Cells were imaged by serial z-scans progressively from bottom to top, in increments of 500 nm. Colocalization of ER-Tracker Red and Newport Green or ZinPyr-1 was measured using Zeiss software [14]. Colocalization was determined by Pearson's correlation coefficient and considered significant when (p ≤ 0.05). Time series measurements of fluorescence intensity were done with image capture at 10 sec intervals, and changes in intensity were measured using Zeiss LSM 510 image analysis software. Fluorescence measurements were background subtracted, normalized to starting values, and expressed as F/Fo.

### Caged IP_3_ experiment

To directly activate IP_3_Rs we used the membrane-permeable UV light-sensitive caged IP_3_ analogue, ci-IP3/PM (D-2,3-*O*-isopropylidene-6-*O*-(2-nitro-4,5-dimethoxy) benzyl-myo-inositol 1,4,5-trisphosphate-hexakis(propionoxymethyl)ester (SiChem. Bremen, Germany) [15,16]. Cells in brain hippocampal slices were simultaneously loaded by incubation with caged IP_3 _and Newport Green for 30 min at 37°C, then washed and incubated for an additional 30 min to allow for complete de-esterification.

IP_3 _was photoreleased by flashes of 364 nm light focused uniformly throughout the field of view.

### Drug Treatments

Thapsigargin (Molecular Probes, Carlsbad, CA), the IP_3_K inhibitor N^2^-(m-trifluorobenzyl),N^6^-(p-nitrobenzyl)purine (Calbiochem, Cat. No. 406170), N,N,N',N'-tetrakis (2 pyridylmethyl) ethylenediamine (TPEN) (Molecular Probes, Carlsbad, CA), the Ins(1,4,5)P_3 _receptor blocker 2-aminoethoxydiphenyl-borate (2-APB), were applied by bath application in HEPES medium.

## Results

### Intracellular Regions of Elevated Zn^2+ ^Co-localize with the Endoplasmic Reticulum

**Figure 1:**
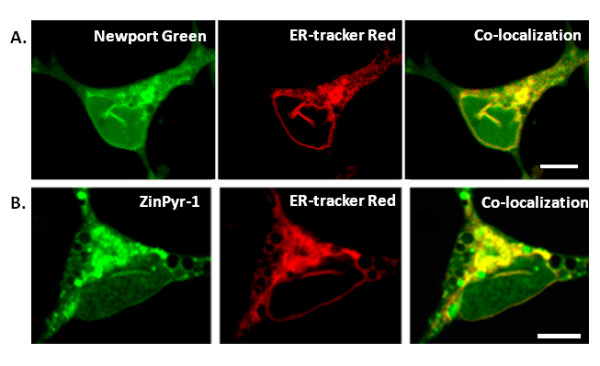
**Co-localization of intracellular Zn^2+ ^and ER fluorescence**. Images of cultured cortical neurons co-labeled with the intracellular fluorescent Zn^2+ ^indicators and a live cell marker for the ER. **A**. Fluorescent Zn^2+ ^indicator Newport Green AM co-localizes with the ER marker ER-Tracker Red. **B**. Fluorescent Zn^2+ ^indicator ZinPyr-1 co-localizes with the ER marker ER-Tracker Red. Cells were incubated with 10 μM of the specified fluorescent Zn^2+ ^indicator and 1 μM ER-Tracker Red then imaged using a LSM510 confocal microscope equipped with a 100x/1.3NA objective.

**Figure 2:**
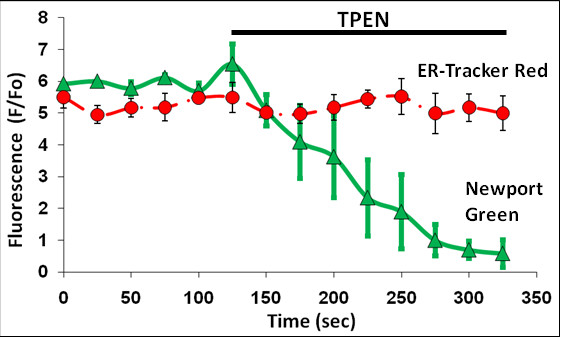
**Effect of Zn^2+ ^chelation**. The graph indicates the fluorescent response of Newport Green (green line) and ER-Tracker Red (red line) in unstimulated neurons upon TPEN (10 μM) exposure, data points represent the mean ± SD of n = 5 trials. The quenching of Newport Green fluorescence with TPEN indicates the presence of free Zn^2+ ^in the basal condition.

Cells labeled with intracellular fluorescent Zn^2+ ^indicators and examined under basal conditions showed consistent regions of elevated fluorescent intensity in the soma and processes, and particularly in a region that was identified as the endoplasmic reticulum by a fluorescent marker for the organelle. The elevated levels of Zn^2+ ^were seen to represent labile or free Zn^2+ ^because they were sensitive to both low and high affinity fluorescent Zn^2+ ^indicators Newport Green (K_D_Zn^2+ ^≈ 10^-6^M) (Figure 1A) and ZinPyr-1(K_D_Zn^2+ ^~ 10^-9^M) (Figure 1B). Before imaging, cells were washed three times with fresh medium to remove excessive fluorescent residues. Another reason for using ZinPyr-1 is that, unlike AM forms of fluorescence indicators, it is highly lipophilic and remains in the organelle, being sequestrated with zinc. These indicators are essentially insensitive to Ca^2+ ^and their fluorescence to Zn^2+ ^are not altered in the presence of Ca^2+ ^[17,18]. Fluorescence was sensitive to quenching by the membrane permeable Zn^2+^-chelator TPEN (K_D_Zn^2+ ^~ 10^-17 ^M) (Figure 2). The same cells were also loaded with ER-tracker Red, a marker for the ER, to determine the localization of the intracellular fluorescent Zn^2+ ^indicators. We performed serial z-scans using confocal microscopy of cortical neurons loaded with ER-tracker Red and either Newport Green or ZinPyr-1 (Figure 1A, B). Both fluorescent Zn^2+ ^indicators showed significant colocalization with the ER Tracker Red. Colocalization was abolished upon exposure to 10 μM TPEN (Figure 2), indicating that the local Zn^2+ ^fluorescence represented free Zn^2+ ^in the basal condition and were located within the lumen of the ER.

### Thapsigargin-Induced Zn^2+ ^Release

**Figure 3:**
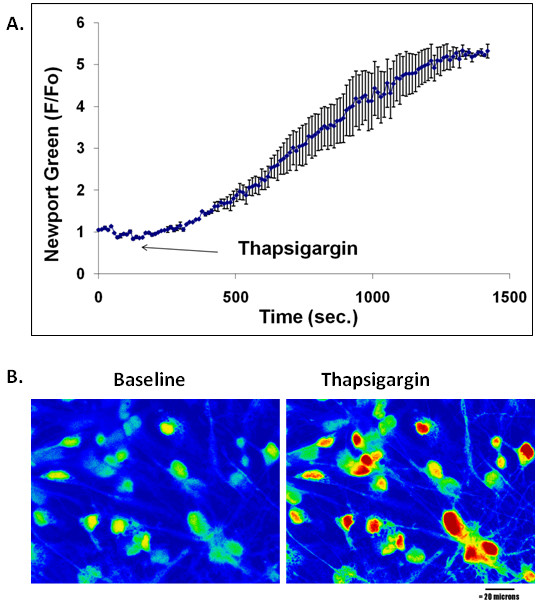
**Thapsigargin-induced Zn^2+ ^release in cultured cortical neurons**. **A**. The graph shows the increases in fluorescence intensity of cells in response to the treatment with thapsigargin (2 μM). Data points represent the mean ± SD of n = 3 trials. Cells were labeled with Newport Green (10 μM). **B**. Representative fluorescent images of cortical neurons loaded with Newport Green before and after exposure to thapsigargin.

**Figure 4:**
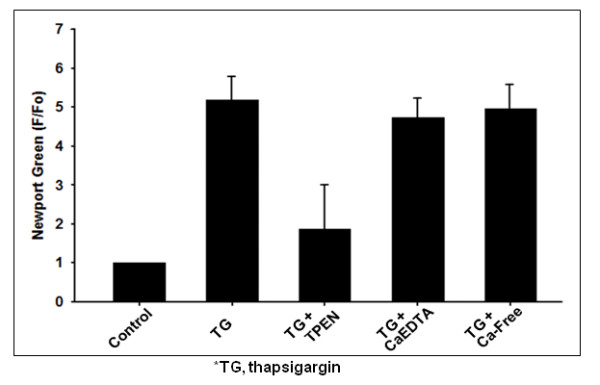
**Summary of Zn^2+ ^transients induced by thapsigargin in cortical neurons**. The bar graphs show peak fluorescence after the treatment with thapsigargin alone (n = 6), thapsigargin plus TPEN (10 μM; n = 6), thapsigargin plus CaEDTA (1 mM; n = 3), and thapsigargin in the medium without added Ca^2+ ^(n = 4). Data points represent the mean ±SD.

To assess the overall dynamics of Zn^2+ ^release from thapsigargin-sensitive stores, cells incubated with Newport Green were exposed to thapsigargin, which inhibits SERCA pump. The application of thapsigargin depletes the ER Ca^2+ ^stores in cells and raises cytosolic Ca^2+ ^concentration. The experiments above suggested that Zn^2+ ^may be also transported into and sequestered in ER. If this was true, the accumulation of Zn^2+ ^by blocking SERCA with thapsigargin should also produce Zn^2+ ^signals. Indeed, exposure to thapsigargin resulted in a gradual increase of cytosolic or intracellular Zn^2+ ^(Figure 3), which was sensitive to the Zn^2+ ^chelator TPEN (Figure 4).

In the next two tests we determined that the source of this thapsigargin-induced Zn^2+ ^increases. One possible source is the influx of extracellular Zn^2+^. To remove extracellular Zn^2+^, we applied a membrane impermeable Zn^2+ ^chelator CaEDTA. As shown in figure 4, thapsigargin-induced Zn^2+ ^rises were unchanged in the presence of CaEDTA (1 mM). Next, we examined the contribution of extracellular Ca^2+ ^on thapsigargin-induced elevation of intracellular Zn^2+^. We found that the removal of extracellular Ca^2+ ^had no effect on the thapsigargin-induced Zn^2+ ^rises (Figure 4). Taken together, these results indicated that thapsigargin-induced increases in intracellular Zn^2+ ^were not dependent on either extracellular Zn^2+^or extracellular Ca^2+^, and were entirely of intracellular origin.

### IP_3_-Induced Intracellular Zn^2+ ^Release

**Figure 5:**
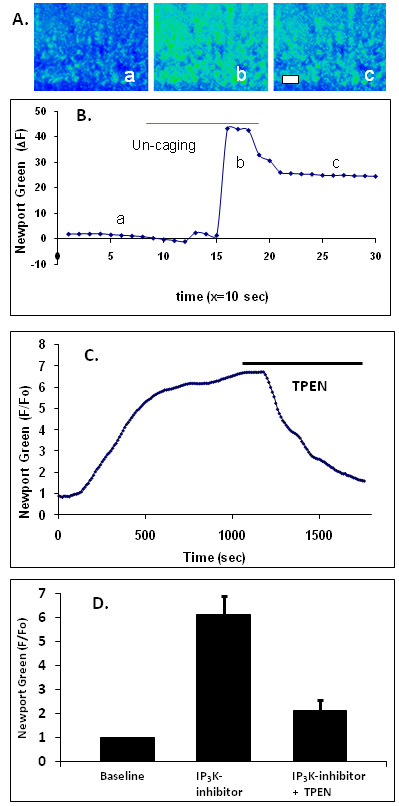
**IP_3 _stimulation results in the release of intracellular Zn^2+^**. **A**. The photo-uncaging of caged IP_3 _(2 μM) in brain hippocampal neurons loaded with the Zn^2+ ^fluorescent indicator Newport Green results in an increase of cytosolic Zn^2+^. Scale bar = 20 μm and the images *a, b, and *corresponding to points in B. **B**. The graph shows a change of fluorescence in response to IP_3 _stimulation. **C**. A change of fluorescence in responses to the treatments of the IP_3_-3K inhibitor N2-(m-trifluorobenzyl),N6-(p-nitrobenzyl)purine (20 μM) and TPEN (10 μM) in cells loaded with Newport Green. **D**. Bar graph shows the average response of Newport Green to the IP_3_K-inhibitor (n = 4). The application of TPEN at the peak of fluorescence resulted in significant (n = 2) fluorescence quenching, demonstrating that the fluorescence elevation was due to increased free Zn^2+^.

The endoplasmic reticulum is a well established site of intracellular Ca^2+ ^storage and release. IP_3 _can trigger the release of Ca^2+ ^from intracellular stores by binding to and activating its receptor (IP_3_R) located on regions of the ER. When IP_3 _binds to and activates IP_3_Rs, the channel portion of the receptor opens and Ca^2+ ^is released from the ER to the cytosol. The experiment was therefore performed utilizing ci-IP3/PM, a cell-permeable form of caged IP_3 _to directly induce Zn^2+ ^release [15,16,19-21]. In neurons loaded with the Zn^2+ ^fluorophore Newport Green and caged IP_3_, IP_3 _uncaging resulted in a rapid increase in intracellular Zn^2+^, which persisted for 30 s and followed by a roughly exponential decay (Figure 5A &5B). This experiment demonstrated that the Zn^2+ ^response was due to the activation of IP_3_ receptors.

A significant route of IP_3 _metabolism is the conversion of IP_3 _into inositol (1,3,4,5)-tetrakisphosphate (Ins(1,3,4,5)P_4_) by the enzyme Ins(1,4,5)P_3 _3-kinase (IP_3_-3Kinase or IP_3_-3K) [22]. Inhibition of the IP_3_-3K has been shown to elevate intracellular levels of IP_3 _by halting its conversion into Ins(1,3,4,5)P4 [23]. Here, to examine the effects of IP_3 _signaling on intracellular Zn^2+ ^dynamics, N^2^-(m-trifluorobenzyl),N^6^-(p-nitrobenzyl)purine, a membrane-permeable inhibitor of IP_3_-3K was employed to confirm the results observed using the caged IP_3_. Upon bath application of the inhibitor, Newport Green fluorescence showed a gradual, significant increase (Figure 5C &5D), supporting an IP_3 _mediated process involved in the release of Zn^2+^.

## Discussion

The major findings of the present study are the following: Neuronal cells maintain a substantial concentration of Zn^2+ ^in ER-like storage, and Zn^2+ ^is released into the cytosol in a thapsigargin- and IP_3_-sensitive manner. These findings suggest a new model of intracellular Zn^2+ ^homeostasis where cellular organelles like the ER act as sites of intracellular Zn^2+ ^storage.

Available data support that intracellular Zn^2+ ^levels can be determined by the interaction of membrane Zn^2+ ^transporters and cytoplasmic Zn^2+ ^buffers [4,10]. In eukaryotic cells the concentration of intracellular Zn^2+ ^has been found to be in the range of a few hundred micromolars [24]. The vast majority of this cellular Zn^2+ ^is, however, protein bound or sequestered into organelles, which results in free cytosolic Zn^2+ ^concentrations being in the picomolar to nanomolar range [10,24,25]. This is consistent with our observations that there is a sharp contrast in Zn^2+ ^fluorescence between ER-like lumen and cytosolic space (Figure 1). The significant concentration of free (labile) Zn^2+ ^present in ER-like lumen suggests the existence of a high intraluminal Zn^2+ ^sequestering activity.

The present study shows that Zn^2+ ^is released from thapsigargin-sensitive and IP_3_R-mediated stores (Figure 3 &5). Thapsigargin, as expected, also induced a Ca^2+ ^transient measured with a fluorescent Ca^2+ ^indicator (data not shown but see [26-28]). Collectively, we suggest that Ca^2+ ^is not the only metal ion that is sequestered in the ER. Zn^2+ ^is released alongside of Ca^2+ ^upon thapsigargin stimulation. We show further that the source of thapsigargin-induced elevation in intracellular Zn^2+ ^is of intracellular origin. The elevation of Zn^2+ ^is independent from either extracellular Ca^2+ ^or extracellular Zn^2+^. These results indicate that Zn^2+ ^homeostasis, like Ca^2+ ^homeostasis, is controlled by IP_3_Rs (Figure 5) that may gate Zn^2+ ^into the cytoplasm, and by thapsigargin sensitive ATPase activity that pumps Zn^2+ ^from the cytoplasm into the ER.

Within the ER, it is known that Ca^2+ ^is buffered by the abundant luminal resident chaperone protein calreticulin which binds Ca^2+^. Although calreticulin was first identified as a Ca^2+ ^binding protein [29], this protein is multifunctional [30] and binds other ions including Zn^2+ ^with multiple binding sites [31-34]. Zn^2+ ^also binds with several other luminal proteins [35]. Just like Ca^2+^, recent work indicates that mitochondria take up cytosolic Zn^2+ ^and that Zn^2+ ^accumulation leads to a loss of mitochondrial membrane potential (see review [36]). There are reports suggesting that thapsigargin/IP_3 _regulate mitochondrial Ca^2+ ^signaling and function [37]. It remains to be studied how thapsigargin and IP_3 _induced Zn^2+ ^release affect mitochondrial function.

While the mechanism(s) that govern Zn^2+ ^trafficking remain elusive, there is little doubt that the intracellular free Zn^2+ ^level must be maintained within a physiological limit. Zn^2+ ^has been shown to activate a number of protein kinases such as protein kinase C, CaMKII, TrkB, Ras and MAP kinase [38-43]. On the other hand, abnormal levels of Zn^2+ ^may lead to either Zn^2+^-induced toxicity or Zn^2+ ^deficiency-induced apoptosis [5]. Therefore, thapsigargin sensitive storage or the ER may function as a source of intracellular free Zn^2+ ^in response to stimuli, and is likely to play an important role in the regulation of intracellular levels of Zn^2+^.

## Competing interests

The authors declare that they have no competing interests.

## Authors' contributions

CS carried out the fluorescence imaging experiments, analysed data, and participated in the experimental design and the preparation of manuscript. YL conceived of the study, and participated in its design, and drafted and prepared manuscript, and coordination. All authors read and approved the final manuscript.
